# The Role of Selected Proteins in the Pathogenesis of Psoriasis

**DOI:** 10.3390/ijms26136475

**Published:** 2025-07-04

**Authors:** Mateusz Matwiejuk, Agnieszka Kulczyńska-Przybik, Hanna Myśliwiec, Adrian Chabowski, Barbara Mroczko, Iwona Flisiak

**Affiliations:** 1 Department of Dermatology and Venereology, Medical University of Bialystok, 15-540 Bialystok, Poland; mateusz.matwiejuk@umb.edu.pl (M.M.);; 2 Department of Neurodegeneration Diagnostics, Medical University of Bialystok, 15-269 Bialystok, Poland; 3 Department of Physiology, Medical University of Bialystok, 15-222 Bialystok, Poland

**Keywords:** psoriasis, elafin, chemerin, nicotinamide phosphoribosyltransferase, visfatin

## Abstract

Psoriasis is a chronic, immune-mediated inflammatory skin disease with complex genetic, environmental, and immunological determinants. Beyond the skin, it affects multiple systems, including the joints and cardiovascular system. A hallmark of psoriasis is an overactivation of the innate and adaptive immune responses, leading to dysregulated cytokine signaling, altered keratinocyte function, and aberrant expression of structural and regulatory proteins. In recent years, growing attention has been given to the skin as a neuro–immuno–endocrine organ, with evidence showing the role of stress-related neuropeptides, UVB-induced immune modulation, and vitamin D signaling in the disease pathogenesis. This review highlights emerging evidence on key multifunctional proteins—elafin, chemerin, and NAMPT (visfatin)—that exert both pro- and anti-inflammatory actions. Although still underexplored, these molecules appear to contribute significantly to the psoriatic microenvironment by modulating inflammation, immunity, and skin barrier function. Their dual roles suggest complex interactions within the cutaneous immune–neuroendocrine network, positioning them as potential biomarkers or therapeutic targets in psoriasis. By integrating insights into classical and emerging mediators, this review aims to provide a comprehensive perspective on the evolving landscape of psoriasis pathophysiology.

## 1. Introduction

Psoriasis is a chronic, immune-mediated, non-contagious, and multidisciplinary disorder, which affects skin, bones, and the vascular system [1]. This disease is a common condition affecting between 0.27% and 11.4% of the adult population worldwide [2]. Psoriasis can develop at any age, including childhood, in which a significant portion (25–40%) of psoriasis cases begin [3]. Early-onset psoriasis can lead to severe physical symptoms and psychological issues later in life [4]. There are various subtypes of psoriasis, with plaque psoriasis being the most common, accounting for nearly 90% of cases. The remaining types of psoriasis include inverse, erythrodermic, pustular, and guttate forms [5]. It is known that chronic plaque psoriasis can affect any part of the body, but the most common areas are the elbows, knees, and scalp [6].

Protein biomarkers are important in the clinical decision-making process, and certain specific biomarkers are indeed commonly used in diagnosing liver diseases (aspartate aminotransferase (AST), alanine aminotransferase (ALT)) and myocardial necrosis (cardiac troponins) [7]. Psoriasis is a complex disease with a multifaceted and discrete pathology due to genetic and immunological alterations resulting from abnormal expression of various regulatory and structural proteins [8]. It has been identified that several proteins play a central role in the pathogenesis of psoriasis, including cytokines, proteases, alarmins, antimicrobial peptides, growth factors, and autoantigens [9]. Plasmacytoid dendritic cells (pDCs) are a type of immune cell that plays a crucial role in the early stages of psoriasis. When activated, pDCs release cytokines like type I interferons (IFN-α and IFN-β) and interleukin-6 (IL-6). Mature pDCs produce inflammatory cytokines such as tumor necrosis factor-alpha (TNF-α), interleukin-12 (IL-12), and interleukin-23 (IL-23). IL-23 drives the differentiation of T helper (Th) cells into Th17 cells, which produce IL-17, IL-21, and IL-22. These cytokines promote inflammation and contribute to the development of psoriatic lesions [10].

Additionally, recent studies have identified several proteins as potential key players in psoriasis pathogenesis, including elafin [11,12], chemerin [13,14], and visfatin (nicotinamide phosphoribosyltransferase—NAMPT) [15,16]; NAMPT and visfatin are synonymous, referring to the same protein. NAMPT (nicotinamide phosphoribosyltransferase) is an enzyme that catalyzes the rate-limiting step in the salvage pathway of nicotinamide adenine dinucleotide (NAD^+^) biosynthesis. The name “visfatin” was later adopted when the protein was identified as a cytokine-like factor secreted by visceral adipose tissue [17].

The skin is vulnerable to environmental stressors like solar radiation and biological, chemical, and physical factors. These stressors initiate local skin responses that impact homeostasis both cutaneously (within the skin) and systemically (throughout the body). A “skin neuro–immuno–endocrine system” that shares mediators with brain pathways regulates skin responses by activating signal transduction pathways and influences the systemic endocrine and immune systems in a context-dependent manner. This intricate skin system is compartmentalized into regulatory units: epidermal, dermal, hypodermal, and adnexal. These units can function independently or together to maintain skin integrity, facilitate adaptation to environmental changes, and prevent disease. These functions are achieved by activating peripheral nerve endings, releasing various signaling molecules (neurotransmitters, hormones, neuropeptides, cytokines, and/or chemokines) into the bloodstream or by priming circulating and resident immune cells. This ultimately helps the body adjust its homeostasis and allostasis to optimally respond to a changing environment [18].

Interestingly, the skin’s ability to perform local endocrine function moves it beyond its traditional role as merely a target for hormones and reveals that the skin itself produces many elements typically associated with the hypothalamus–pituitary–adrenal (HPA) axis—a major stress response system in the body. The skin expresses and produces critical HPA axis components, including corticotropin-releasing hormone (CRH), urocortin, and proopiomelanocortin (POMC), along with HPA axis derivatives like ACTH, α-MSH, and β-endorphin. This local production of components, coupled with the presence of their corresponding receptors on the same cells, suggests paracrine (acting on nearby cells) or autocrine (acting on the same cell) mechanisms of action. Beyond HPA axis components, the skin also produces other hormones like vitamin D3, PTH-related protein (PTHrP), catecholamines, and acetylcholine. The regulation of these hormones by environmental stressors such as UV light underscores their role in the skin’s response to stress [19].

Ultraviolet B (UVB; wavelength 290–320 nm) can stimulate the production of CRH peptides, contrary to dexamethasone, which inhibits CRH peptide production [20]. These endocrine mediators and their receptors are organized into distinct dermal and epidermal units. This compartmentalization allows for precise and field-restricted control of their activity [19]. This skin’s neuroendocrine system communicates both internally (within the skin) and with the systemic level through humoral (blood-borne) and neural pathways. This link may trigger various changes, including vascular, immune, or pigmentary responses, and can directly counteract harmful agents or neutralize local reactions. The overarching suggestion is that the skin’s neuroendocrine system plays a crucial role in preserving and maintaining the skin’s structural and functional integrity and, by extension, systemic homeostasis throughout the body [19].

Numerous studies have demonstrated the pivotal role of various proteins in the pathogenesis of diverse skin conditions, including psoriasis. However, a comprehensive understanding of these molecular mechanisms remains elusive. This narrative review aims to elucidate the potential role of proteins in the development and progression of psoriasis.

## 2. Materials and Methods

A comprehensive literature search was conducted in PubMed and Google Scholar, covering the period from 1957 to January 2025, without date restrictions. The primary aim was to identify and analyze the role of selected proteins—NAMPT (visfatin), chemerin, and elafin—in the pathogenesis of psoriasis, along with broader aspects of skin neuro–immuno–endocrine interactions. The search strategy was expanded to include the literature related to the following areas: neuro–immuno–endocrine functions of the skin, stress-related neuropeptides, the impact of UVB radiation on immune pathways in psoriasis, and the role of vitamin D in skin immunity and homeostasis. The following Medical Subject Headings (MeSH) and keyword combinations were used: “chemerin in psoriasis”, “elafin in psoriasis”, “visfatin in psoriasis”, “NAMPT in psoriasis”, “skin neuro–immuno–endocrine system”, “neuropeptides and psoriasis”, “UVB and psoriasis”, “vitamin D and psoriasis” and “oxidative stress and psoriasis”. The search included original studies in humans and animal models relevant to molecular, immunological, and clinical aspects of psoriasis. Non-English publications, studies of low clinical relevance, case reports, and duplicates were excluded. After de-duplication, titles and abstracts were independently screened by three reviewers (M.M., A.K-P., H.M.). Disagreements were resolved through consensus between the same reviewers. Full texts of eligible articles were then assessed in detail.

A medical literature search of PubMed and Google Schoolar (1957–present), conducted in the winter of 2025, was performed using appropriate terms without date limitations. The main object of the research was to identify the role of selected proteins like NAMPT (visfatin), chemerin, and elafin in the pathogenesis of psoriasis. Medical subject headline terms included “chemerin in psoriasis”, “elafin in psoriasis”, “visfatin in psoriasis”, and “NAMPT in psoriasis”. Non-English publications, papers with low clinical significance, and duplicated publications were excluded from the analysis. Originally, human and animal studies were included in this narrative review. The results of the search strings were combined together, and duplicates were removed. Afterwards, the titles and abstracts of the searched studies were independently screened by three reviewers (M.M., A.K-P. and H.M.) in order to identify relevant articles that addressed the review subject. Disagreements between reviewers were resolved by mentioned reviewers (A. K-P., H.M.). Finally, the selected eligible articles were fully reviewed.

## 3. Discussion

### 3.1. Elafin

Elafin, also called skin-derived anti-leukoprotease (SKALP), is derived from pre-elafin through proteolytic cleavage, often mediated by mast cell tryptase [21,22]. Epithelial cells and keratinocytes are the main sources of elafin. Elafin expression is upregulated by pro-inflammatory cytokines like interleukin-1 (IL-1) and tumor necrosis factor-α (TNF-α), particularly in conditions like psoriasis [11,23]. Elafin acts as a potent inhibitor of various serine proteases, including neutrophil elastase, proteinase 3, and vascular elastase. Interestingly, elafin, as a potent inhibitor of neutrophil elastase, can also be cleaved by the same enzyme [24,25]. Elafin plays a crucial role in protecting tissues from damage caused by inflammation. It helps to maintain tissue integrity by inhibiting the destructive effects of proteases. In addition, elafin exhibits antimicrobial properties against a broad spectrum of pathogens, such as bacteria, viruses, parasites, and fungi; therefore, these antimicrobial properties contribute to the body’s defense against infection [26,27].

#### Elafin’s Role in the Pathogenesis of Psoriasis

The findings of Nakane et al. [17] suggested that in psoriatic skin, elafin plays a crucial role in the formation and structure of the cell envelope. Elafin is minimally expressed and not typically detected on the cell envelope (CE) in normal skin, contrary to psoriatic skin, where elafin is highly expressed and can be a major component of the CE. Lowicryl K11M (polar) resin allowed for better detection of surface antigens like elafin on the CE. On the other hand, lowicryl HM20 (apolar) was less effective for detecting surface antigens. While some elafin was released extracellularly to contribute to skin barrier function, a significant portion remained intracellularly and became integrated into the CE. This incorporation of elafin into the CE may contribute to the thickened, hyperproliferative epidermis characteristic of psoriasis. In this result, elafin shows promise as a potential biomarker for monitoring disease activity and treatment response in psoriasis. In addition, understanding the role of elafin in psoriatic skin may lead to the development of new therapeutic strategies targeting elafin expression or function [17] (Table 1).

Holmannova et al. [28] stated that patients with psoriasis exhibited significantly higher serum levels of elafin compared with healthy controls. Increased expression of elafin in psoriasis may contribute to both inflammation and alterations in skin barrier function. The study also suggested possible correlations between elafin levels and various factors, including age, the body mass index (BMI), the psoriasis area and severity index (PASI) score, and certain metabolic parameters. Alterations in elafin levels could be used to monitor how well a patient is responding to treatment [28] (Table 1).

Elgharib et al. [11] revealed that elafin levels in the serum were significantly higher in psoriasis patients (including smokers) compared with healthy controls (including non-smokers). Firstly, elafin levels were correlated with psoriasis severity, as measured by the PASI score and with inflammatory markers like C-reactive protein (CRP) and the erythrocyte sedimentation rate (ESR), indicating a strong association between inflammation and disease severity. Secondly, patients with a positive family history of psoriasis had significantly higher elafin levels. Summing up, higher elafin levels are associated with increased disease severity and inflammation. Elafin could be a potential therapeutic target for psoriasis, and even more, its levels could serve as a biomarker for disease activity and treatment response. Additionally, according to the study, smoking cessation might be beneficial for psoriasis patients [11] (Table 1).

Albarazenji et al. [29] further solidified the role of elafin as a biomarker for psoriasis. The authors found a strong correlation between serum elafin levels and the severity of psoriasis, as measured by the PASI score. Moreover, they observed a significant decrease in elafin levels following successful narrowband-ultraviolet B (NB-UVB) treatment, which could be used to assess the effectiveness of treatments like NB-UVB therapy [29] (Table 1).

Alghonemy et al. [30] further supported the role of elafin as a potential biomarker for psoriasis. Psoriasis patients exhibited significantly higher serum elafin levels (6.09 ± 8.91) compared with healthy controls (0.40 ± 0.35), with *p* less than 0.001, and its levels were positively correlated with PASI scores, indicating a link between elafin expression and disease severity (*r* = 0.467 and *p* < 0.001). Elevated levels of inflammatory markers like ESR and CRP were observed in psoriasis patients, with *p* less than 0.001, further supporting the inflammatory nature of the disease. In conclusion, the collective findings from these studies highlighted the potential of elafin as a biomarker and therapeutic target for psoriasis [30] (Table 1).

The research of Elghetany et al. [31] further strengthened the role of elafin as a potential biomarker for psoriasis. First of all, psoriasis patients exhibited significantly higher serum elafin levels compared with healthy controls (*p* < 0.001). Secondly, elafin levels were positively correlated with PASI scores, indicating a link between elafin expression and disease severity. Thirdly, elevated levels of inflammatory markers like ESR and CRP were observed in psoriasis patients, further approving the inflammatory background of this dermatosis. Finally, smoking and a positive family history of psoriasis were also associated with increased serum elafin levels [31] (Table 1).

Alkemade et al. [32] also showed that elafin correlated strongly with the PASI score. Serum elafin showed a more significant and reliable correlation with the clinical course compared with urine elafin. Although urine elafin can also be used as a biomarker, it is less sensitive and specific than serum elafin. Serum elafin levels may serve as a useful marker for assessing disease severity and monitoring treatment response to cyclosporine A. Tracking changes in elafin levels could help predict treatment efficacy. In summary, targeting elafin may offer novel therapeutic approaches for psoriasis [32] (Table 1).

Tanaka et al. [33] presented various elafin serum levels in different types of psoriasis. In generalized pustular psoriasis, significantly higher elafin levels were observed compared with normal controls. In pustulosis palmoplantaris (PPP), no significant difference was spotted in elafin levels compared with normal controls. Interestingly, despite high local elafin expression in non-pustular regions of PPP and generalized pustular psoriasis, serum elafin levels may be lower. This suggests that systemic elafin levels may not accurately reflect local skin inflammation in these conditions. On the contrary, increased local inflammation and neutrophil activation in pustular regions may lead to rapid consumption of elafin, limiting its release into the systemic circulation. The inflammatory process in pustular psoriasis may disrupt the normal release of elafin from keratinocytes into the bloodstream. Finally, the specific inflammatory mediators and signaling pathways involved in pustular psoriasis may differentially regulate elafin expression compared with other types of psoriasis [33] (Table 1).

Nonomura et al. [34] showed different locations of elafin in various affected locations of psoriatic skin. In the non-lesional psoriatic epidermis, there was focal expression in the subcorneal layer. In the involved psoriatic epidermis, there was markedly increased expression in the upper spinous and granular layers; maximal expression was marked near subcorneal microabscesses. In pustular psoriasis, there was increased expression in the spinous layers under subcorneal pustules. Increased expression of elafin mRNA in psoriatic epidermis suggests a role in epidermal differentiation and barrier function. The localization of elafin expression near inflammatory cells and subcorneal microabscesses indicates a potential role in the inflammatory response [34] (Table 1).

Schalkwijk et al. [35] revealed strong elafin expression in the upper suprabasal layers of the psoriatic epidermis, especially in the upper parakeratotic layers and stratum corneum. Firstly, strong expression of elafin in the upper suprabasal layers suggests a role in epidermal differentiation and barrier function. Secondly, the cornified envelope’s staining indicates elafin’s potential role in cross-linking envelope proteins and maintaining skin barrier integrity [35] (Table 1).

Kuijpers et al. [36] suggested that mutations in the coding region of the elafin gene are not a major determinant of pustular psoriasis. A single nucleotide polymorphism (SNP) was identified in exon 1 of the elafin gene, resulting in a threonine-to-alanine substitution in the signal peptide. A dinucleotide repeat polymorphism was found in the promoter region of the elafin gene. Neither of these polymorphisms was associated with plaque-type psoriasis, pustular psoriasis, or psoriasis in general. While genetic factors play a role in psoriasis, mutations in the coding region of the elafin gene do not appear to be a major cause of pustular psoriasis. The decreased elafin activity in the psoriatic skin observed in pustular psoriasis may be due to other mechanisms, such as epigenetic factors or post-translational modifications [36].

To sum up, elafin inhibits neutrophil elastase and suppresses NF-κB-mediated expression of pro-inflammatory cytokines such as TNF-α and IL-8, thereby reducing inflammation and protecting tissues. Its ability to rebalance the protease/anti-protease axis suggests a potential role in mitigating inflammation-driven pathology in diseases like psoriasis. Current clinical trials involving elafin focus on its use in reducing postoperative inflammation, particularly in surgical and transplant settings. These studies indicate that elafin may have broader therapeutic applications in inflammatory diseases. While no published research to date has directly investigated elafin as a treatment target for psoriasis, its anti-inflammatory properties and emerging clinical relevance highlight its potential for future therapeutic strategies in psoriatic disease.

**Table 1:** Summary of the studies on elafin role in psoriasis.

Author	Year	Population	Key Observation
Elafin in Psoriasis			
Nakane et al. [17]	2002	6 patients with psoriasis	Elafin may contribute to the thickened, hyperproliferative epidermis characteristic of psoriasis.
Holmannova et al. [28]	2020	85 patients with psoriasis	Elafin levels were correlated with psoriasis severity, as measured by the PASI score and with inflammatory markers CRP and ESR.
Elgharib et al. [11]	2019	N1—26 healthy people N2—26 patients with psoriasis	Higher elafin levels in serum were associated with increased disease severity and inflammation.
Albarazenji et al. [29]	2021	N1—30 healthy people N2—30 patients with psoriasis	Elafin could be used to assess the effectiveness of treatments like NB-UVB therapy.
Alghonemy et al. [30]	2020	N1—30 healthy people N2—60 patients with psoriasis	Elafin level was positively correlated with PASI scores, indicating a link between elafin expression and disease severity.
Elghetany et al. [31]	2021	N1—45 healthy people N2—45 patients with psoriasis	Smoking and a positive family history of psoriasis were also associated with increased serum elafin levels.
Alkemade et al. [32]	1995	N1—15 healthy people N2—6 patients with psoriasis	Elafin correlated strongly with the PASI score.
Tanaka et al. [33]	2000	9 patients with pustulosis palmoplantaris; 3 patients with generalized pustular psoriasis	Increased local inflammation and neutrophil activation in pustular regions may lead to rapid consumption of elafin.
Nonomura et al. [34]	1994	N2—5 patients with psoriasis	Increased expression of elafin mRNA in psoriatic epidermis suggested a role in epidermal differentiation and barrier function.
Schalkwijk et al. [35]	1993	N1—7 healthy people N2—9 patients with psoriasis	Strong elafin expression was present in the upper suprabasal layers of the psoriatic epidermis.
Kuijpers et al. [36]	1998	30 patients with chronic plaque psoriasis; 12 patients with pustulosis palmoplantaris; 1 patient with acrodermatitis continua of Hallopeau; 1 patient with generalized pustular psoriasis von Zumbusch; 1 patient with non-generalized pustular psoriasis	Mutations in the coding region of the elafin gene did not appear to be a major cause of pustular psoriasis.

Abbreviations: N1—control group, N2—study group, PASI—psoriasis area and severity index, NB-UVB—narrowband-ultraviolet B.

### 3.2. Chemerin

In 1997, chemerin was primarily described as a retinoid-responsive gene, which is present in psoriatic skin [37].

According to the most recent data, chemerin is an inflammatory tissue protein produced by fibroblasts, mast cells, and endothelial cells [38]. Chemerin is primarily produced in the visceral adipose tissue (VAT), placenta, and liver. It is also produced to a lesser extent in the lungs, heart, ovaries, kidneys, and pancreas [39]. Chemerin has a variety of functions in the body, including attracting immune cells to sites of inflammation, regulating energy metabolism and adipogenesis, and promoting the growth of new blood vessels [38].

Most circulating chemerin exists as inactive prochemerin, which undergoes a proteolytic process to become bioactive chemerin [40].

Chemerin is abundantly expressed in the skin, making it a potential first-line defense against pathogens. Proteases secreted by *S. aureus* can cleave inactive prochemerin into its active form, triggering its antimicrobial and immunomodulatory effects. Chemerin interacts with its receptor, CMKLR1, to modulate immune responses [41].

#### Chemerin Role in the Pathogenesis of Psoriasis

Kong et al. [42] noted that chemerin can enhance keratinocyte proliferation, increase the production of inflammatory cytokines, and activate the MAPK signaling pathway, all of which contribute to the development of psoriasis. Furthermore, they found that neutralizing chemerin with an antibody can reduce epidermal proliferation and inflammation in a mouse model of psoriasis. These findings suggest that chemerin could be a potential target for treating psoriasis [42] (Table 2).

Borsky et al. [43] found that chemerin levels were significantly higher (11,799.0 (9255.8–14,037.8)) in individuals with psoriasis compared with healthy controls (8592.0 (5045.8–12,569.3)) (*p* < 0.05). While their study did not find a direct correlation between chemerin levels and the severity of psoriasis (the PASI score), they did observe a significant negative relationship between the two components (Spearman’s rho = −0.424, *p* < 0.05). Additionally, the authors found a significant positive correlation between chemerin levels and CRP levels, indicating a potential link between inflammation and chemerin production (rho = 0.543, *p* < 0.01) [43] (Table 2).

Connection between chemerin levels and psoriasis severity was also studied by Coban et al. [44]. The study involved 35 patients with psoriasis who received methotrexate, a systemic treatment for psoriasis. Before treatment, patients had significantly higher chemerin levels (125.28 ng/mL) compared with the control group (100.04 ng/mL). Following methotrexate treatment, patients showed significant improvement in their PASI score, and their chemerin levels dropped significantly (3.7 ng/mL). The decrease in chemerin paralleled reductions in high-sensitive-CRP (hs-CRP) (from 2.41 ± 1.59 ng/mL to 1.99 ± 0.96 ng/mL), another marker of inflammation. This study suggests that chemerin may be involved in psoriasis and that reducing chemerin levels could be beneficial for treatment [44] (Table 2).

Aksu et al. [45] investigated the relationship between chemerin and cardiovascular health in patients with psoriasis. The researchers found that chemerin levels were positively correlated with several cardiovascular risk factors, including age, the body mass index, blood pressure, waist circumference, and markers of cardiac dysfunction (early diastolic peak velocity of mitral inflow/early diastolic mitral annular velocity (E/E’) and epicardial fat tissue). Conversely, chemerin levels were negatively correlated with markers of better cardiovascular function (early diastolic mitral annular velocity (E’), early diastolic mitral annular velocity/late diastolic mitral annular velocity (E’/A’), and flow-mediated dilatation). The study also found that patients with psoriasis had significantly higher chemerin levels compared with healthy controls (332 ± 73 ng/mL vs. 301 ± 60 ng/mL; *p* = 0.04). These findings suggest that chemerin may be a useful biomarker for identifying individuals with psoriasis who are at increased risk for cardiovascular disease. Additionally, targeting chemerin may be a potential therapeutic strategy for preventing cardiovascular complications in patients with psoriasis [45] (Table 2).

Similarly to the previously mentioned studies, Tekely et al. [46] evaluated the relationship between chemerin and psoriasis. First, patients with psoriasis had significantly elevated serum chemerin levels compared with healthy controls, (206.93 ng/mL vs. 174.54 ng/mL) (*p* = 0.0003). Second, no correlation was found between serum chemerin levels and the severity of psoriasis measured by the PASI score. Third, chemerin levels showed a positive correlation with inflammatory markers such as CRP and ESR. Fourth, chemerin levels were positively correlated with triglyceride levels and negatively correlated with high-density lipoprotein (HDL) cholesterol. These findings suggest that chemerin may contribute to the inflammatory and metabolic disturbances associated with psoriasis [46] (Table 2).

Zeid et al. [47] reported elevated chemerin levels both in plasma and skin of psoriatic patients. Interestingly, higher chemerin levels in serum and skin tissue are more common in recent-onset psoriasis compared with long-standing cases. Despite its recognized role in inflammation, chemerin does not seem to be linked to traditional cardiovascular risk factors like metabolic syndrome. Both tissue and plasma chemerin levels are elevated in psoriasis patients, with significantly higher levels in psoriatic lesions compared with normal skin (*p* < 0.001 both). The mean amounts of chemerin in psoriatic skin lesions, non-lesioned skin of psoriatic patients, and in normal skin of controls were 28.1 ± 9.28, 13.34 ± 4.23, and 5.22 ± 1.98 ng/mg, respectively (*p* < 0.001). The average plasma levels of chemerin in patients dealing from psoriasis and controls were 52.45 ± 11.83 and 9.4 ± 1.6 μg/mL, respectively. Moreover, psychic stress appears to contribute to increased tissue chemerin levels in psoriasis patients (regression coefficient—(RC) 16.9 μg/mL, 95% CI 11.33–22.59, *p* < 0.001 and RC 9.1 μg/mL, 95% CI 3.77–14.38, *p* = 0.002, respectively). Summing up, chemerin could be a potential biomarker for early-stage psoriasis diagnosis and monitoring disease activity. Moreover, targeting chemerin or its signaling pathways could be a new modality for psoriasis [47] (Table 2).

Al-Sheikh et al. [48] also presented research showing increased levels of chemerin in psoriatic patients. Significantly elevated plasma levels of chemerin were found in patients suffering from psoriasis as compared with healthy people (351.2 ± 68.2 vs. 210.6 ± 46.5, respectively) (*p* < 0.001). Additionally, a strong positive correlation was observed between the PASI score and chemerin levels, suggesting that higher disease severity is associated with increased chemerin levels. This was accompanied by increased carotid intima-media thickness (CIMT) and epicardial fat thickness (EFT), which are markers of subclinical atherosclerosis, highlighting its potential role in the development of atherosclerosis in psoriasis patients. Moreover, a positive correlation revealed that the PASI score was positively linked to chemerin, EFT and CIMT (*p* < 0.001). In conclusion, chemerin could serve as a valuable biomarker for assessing cardiovascular risk in psoriasis patients. Modulating chemerin or its associated signaling pathways may represent a promising therapeutic approach for mitigating cardiovascular risk in patients with psoriasis [48] (Table 2).

Wang et al. [49] observed that chemerin might play a role in regulating the balance between Th9 and Treg cells in psoriasis patients. Both chemerin and its receptor, ChemR23, were found to be upregulated in the peripheral blood of psoriasis patients compared with healthy controls (all *p* < 0.05). In addition, psoriatic patients exhibited a skewed Th9/Treg balance, with an increased ratio of Th9 to Treg cells in comparison with healthy people (all *p* < 0.05). Furthermore, treatment of CD4+ T cells with chemerin led to increased production of pro-inflammatory cytokines IL-6, IL-9, and IL-17 (all *p* < 0.05). Chemerin subsequently exacerbated the Th9/Treg imbalance by promoting Th9 cell differentiation. In fact, silencing ChemR23 reversed the effects of chemerin on T cell cytokine production and Th9/Treg balance. Therefore, targeting chemerin or its signaling pathway could be a potential therapeutic strategy for psoriasis. Measuring chemerin levels and Th9/Treg balance could serve as biomarkers for monitoring disease activity and treatment response [49] (Table 2).

**Table 2:** Summary of the studies on chemerin role in psoriasis.

Author	Year	Population	Key Observation
Chemerin in Psoriasis			
Kong et al. [42]	2023	Psoriasis-like inflammatory cell model and imiquimod (IMQ)-induced mouse model	Neutralizing chemerin could reduce epidermal proliferation and inflammation in a mouse model of psoriasis.
Borsky et al. [43]	2021	N1—22 healthy people N2—28 psoriatic patients	A direct correlation between chemerin levels and the PASI score was not found.
Coban et al. [44]	2016	N1—50 healthy people N2—35 psoriatic patients	The decrease in chemerin paralleled reductions in high-sensitive-CRP.
Aksu et al. [45]	2017	N1—32 healthy people N2—60 psoriatic patients	Chemerin may be a useful biomarker for identifying individuals with psoriasis who are at increased risk for cardiovascular disease.
Tekely et al. [46]	2018	N1—40 healthy people N2—66 psoriatic patients	There was no correlation between chemerin levels and the severity of the psoriasis—PASI score.
Zeid et al. [47]	2012	N1—10 healthy people N2—20 psoriatic patients	Higher chemerin levels in plasma and skin tissue were more common in recent-onset psoriasis compared with long-standing cases.
Al-Sheikh et al. [48]	2019	N1—40 healthy people N2—50 psoriatic patients	Positive correlation revealed that the PASI score was positively linked to the level of chemerin in plasma, EFT, and CIMT.
Wang et al. [49]	2019	N1—20 healthy people N2—25 psoriatic patients	Chemerin subsequently exacerbated the Th9/Treg imbalance by promoting Th9 cell differentiation.

Abbreviations: CIMT—carotid intima-media thickness, EFT—epicardial fat thickness.

### 3.3. NAMPT (Visfatin)

In 1957, the identification of nicotinamide phosphoribosyltransferase (NAMPT) as an enzyme involved in the biosynthesis of NAD was reported [50]. NAMPT is secreted by visceral adipose tissue, and its circulating levels have been shown to correlate with the adiposity in obese individuals. This association led to its designation as “visfatin”, identifying it as an adipokine with potential roles in regulation of metabolism and inflammation [51]. Visfatin, a protein synthesized in response to inflammatory signals like tumor necrosis factor α (TNFα), IL-1, and IL-6, exhibits pro-inflammatory properties. Visfatin induces expression of costimulatory molecules (CD80, CD40, and intercellular adhesion molecule 1 (ICAM-1)) on T cells, which are essential for their activation and subsequent immune response [52].

#### NAMPT (Visfatin) Role in the Pathogenesis of Psoriasis

Gerdes et al. [53] demonstrated that serum visfatin levels are significantly higher in individuals with psoriasis compared with healthy controls. Moreover, visfatin levels were positively correlated with the BMI in the psoriasis patient group, but not in the control group. This suggests that obesity, which is often associated with increased inflammation, may further amplify visfatin production in psoriasis patients [53] (Table 3).

Ismail et al. [54] further solidified the link between visfatin and psoriasis severity. Similar to previous studies, the authors found significantly higher serum visfatin levels in psoriasis patients (62.2 ± 39.4) compared with healthy controls (21.3 ± 15.3) (*p* < 0.0001). Visfatin levels were positively correlated with the PASI, which indicated that higher visfatin levels may be associated with more severe disease (*p* = 0.037). Visfatin levels also showed a positive correlation with the duration of psoriasis, which suggested that chronic inflammation, as observed in long-standing psoriasis, may further elevate visfatin levels. Unlike some other studies, the researchers did not find a significant association between visfatin levels and the BMI, suggesting that obesity might not be a major factor influencing visfatin levels in psoriasis (*p* = 0.397). These findings support the hypothesis that visfatin plays a role in the inflammatory processes underlying psoriasis [54] (Table 3).

Mercurio et al. [15] provided a fascinating insight into the role of NAMPT-mediated NAD+ metabolism in the pathogenesis of psoriasis. The study found increased NAD+ levels in the lesional skin of psoriasis patients, which is associated with high levels of NAMPT—an enzyme involved in NAD+ synthesis. NAMPT expression was significantly upregulated in psoriatic skin in response to pro-inflammatory cytokines like IL-17A. Intracellular NAMPT, by increasing NAD+ levels, promoted keratinocyte proliferation and inhibited their terminal differentiation, contributing to the thickened epidermis observed in psoriasis. NAMPT-mediated NAD+ boost synergized with psoriasis-related cytokines to upregulate inflammatory chemokines, leading to increased recruitment of neutrophils and Th1/Th17 cells. Extracellular NAMPT, released by keratinocytes and fibroblasts, acted on endothelial cells, inducing their proliferation, migration, and expression of adhesion molecules and chemokines, further promoting inflammation. These findings suggest that NAMPT-mediated NAD+ metabolism plays a crucial role in amplifying the inflammatory response in psoriasis [15] (Table 3).

Xie et al. [55] highlighted the potential of NAMPT as a biomarker for distinguishing psoriatic lesions from normal skin. The study demonstrated that NAMPT expression was significantly upregulated in psoriatic lesions compared with both non-lesional and healthy skin. Furthermore, NAMPT was identified as a potential biomarker capable of differentiating lesional from non-lesional skin in patients with psoriasis. These findings provide important insights into the molecular mechanisms underlying psoriasis and may support the development of more accurate diagnostic tools and targeted therapeutic approaches [55] (Table 3).

Hau et al. [56] shed light on the role of visfatin in the upregulation of antimicrobial peptides in psoriasis. Visfatin was found to stimulate the production of antimicrobial peptides (AMPs) such as cathelicidin (CAMP), beta-defensin-2 (hBD-2), beta-defensin-3 (hBD-3), and S100A7 in human keratinocytes. Subsequently, the increased production of AMPs by visfatin might contribute to the inflammatory response in psoriasis by promoting immune cell recruitment and activation. In a mouse model of psoriasis induced by imiquimod, visfatin treatment led to an increase in expression of AMPs in the skin, further supporting its role in psoriasis pathogenesis. Summing up, these findings suggest that visfatin may be a potential therapeutic target for psoriasis. By inhibiting visfatin-mediated AMP production, it may be possible to reduce inflammation and improve symptoms in psoriasis patients [56] (Table 3).

**Table 3:** Summary of the studies on NAMPT (visfatin) role in psoriasis.

Author	Year	Population	Key Observation
NAMPT (Visfatin) Role in the Pathogenesis of Psoriasis			
Gerdes et al. [53]	2012	N1—80 healthy people N2—79 psoriatic patients	Visfatin levels were positively correlated with the BMI in the psoriasis patient group, but not in the control group.
Ismail et al. [54]	2012	N1—42 healthy people N2—46 psoriatic patients	Visfatin levels were positively correlated with the PASI, which indicated that higher visfatin levels may be associated with more severe disease.
Mercurio et al. [15]	2021	N1—10 healthy people N2—25 psoriatic patients	Intracellular NAMPT, by increasing NAD+ levels, promoted keratinocyte proliferation and inhibited their terminal differentiation, contributing to the thickened epidermis observed in psoriasis.
Xie et al. [55]	2014	N1—21 healthy people N2—33 psoriatic patients (lesional skin), 28 psoriatic patients (non-lesional skin)	NAMPT gene was found to be significantly upregulated in psoriatic lesions compared with non-lesional skin and healthy skin.
Hau et al. [56]	2013	N1—8 healthy people N2—8 psoriatic patients Animals: Female BALB/c mice aged 6 to 8 weeks, murine	The increased production of AMPs by visfatin might contribute to the inflammatory response in psoriasis by promoting immune cell recruitment and activation.

Abbreviations: NAMPT—nicotinamide phosphoribosyltransferase, NAD—nicotinamide adenine dinucleotide, AMPs—antimicrobial peptides.

### 3.4. The Skin as a Neuro–Immuno–Endocrine Organ in Psoriasis

Haimakainen et al. [57] showed that corticotropin-releasing hormone receptor type 1 (CRH-R1) expression was significantly increased in lesional skin psoriasis (*p*  =  0.02). The percentage of CRH-R1+ mast cells was significantly increased in the lesional skin of patients with psoriasis. Interestingly, it has been indicated that exposure to UVB radiation at specific doses of 90 mJ/cm^2^ (*p* = 0.041) and that 120 mJ/cm^2^ of UVB exposure significantly decreased CRH-R1 expression (*p* = 0.039) [57]. On the other hand, Zhou et al. [58] demonstrated that expression of CRH and its receptor CRH-R1 is lower in the lesional skin of chronic plaque psoriasis compared with both the perilesional psoriatic skin and normal control skin. The authors also found that CRH reduced expression of IL-18, a pro-inflammatory cytokine, in human keratinocyte cell line cells. This effect is mediated through the CRH-R1 receptor and involves the mitogen-activated protein kinase signaling pathway. The findings suggest the presence of an “aberrant cutaneous CRH/CRH-R1 system” within psoriatic lesions. This aberrant system is actively involved in the development of psoriasis. Consequently, the study proposes that the CRH/CRH-R1 system warrants further investigation as a potential target for new therapeutic strategies in treating psoriasis. The idea is that modulating this system could help correct the observed aberrancy and alleviate disease symptoms [58]. According to Galimova et al. [59], CRH may have pro- and anti-inflammatory functions by being involved in the etiology of inflammatory skin disorders. Stimulation of CRHR1 by CRH leads to activation diverse signaling pathways that control proliferation, differentiation, apoptosis, and anti- or pro-inflammatory activities of skin cells. The proteins linked to the corticotropin-releasing hormone–proopiomelanocortin (CRH–POMC) system show functional association. Among these proteins, proopiomelanocortin (POMC) and agouti-signaling protein (ASIP) for the melanocortin 1 receptor (MC1R) and tyrosinase (TYR) and tyrosinase-related protein 1 (TYRP1) for dopachrome tautomerase (DCT) demonstrated the highest scores. To conclude, this study showed the associations between MC1R and DCT polymorphisms and psoriasis in the Tatar population, suggesting that these genetic variants may contribute to a predisposition to psoriasis [59].

It has been shown that expression of POMC is increased in both lesional and non-lesional psoriatic skin compared with healthy controls. Skin expression levels of the POMC gene and POMC/corticotropin-releasing hormone (CRH) peptides are not static but are determined by such factors as the physiological changes associated with the hair cycle (highest in the anagen phase), ultraviolet radiation (UVR) exposure, immune cytokine release, or the presence of cutaneous pathology [20]. Additionally, patients with psoriasis exhibit significantly higher concentrations of beta-endorphin in the blood compared with healthy controls. Notably, elevated beta-endorphin levels correlate with disease severity and the extent of skin involvement, particularly in cases with widespread psoriatic lesions [20].

Met-enkephalin and Leu-enkephalin are endogenous opioid peptides, meaning they are naturally produced by the body. They are derived from a precursor protein called proenkephalin (PENK). The skin itself is a significant site of proenkephalin gene and protein expression. These enkephalins are found in various skin cells, including keratinocytes (the main cells of the epidermis) and fibroblasts, as well as in the outer root sheath of hair follicles and eccrine glands. Enkephalins and their receptors (like delta-opioid receptors, DOR, and the zeta (ζ) opioid receptor—also known as opioid growth factor receptor) are crucial for regulating various skin functions, including cell proliferation, differentiation, and immune responses. They are believed to tonically inhibit DNA synthesis in the epidermis [60]. A key finding is that Met/Leu-enkephalin expression is increased in psoriatic skin lesions. This upregulation of PENK and its derived peptides in psoriatic keratinocytes has been consistently reported. Enkephalins normally inhibit keratinocyte proliferation; their increased levels in psoriasis might represent a failed attempt by the body to suppress the excessive keratinocyte growth characteristic of the disease. This is suggested by studies where topical treatments (like calcipotriol and mometasone furoate) that improve psoriasis also lead to a decrease in Met-enkephalin levels in parallel with clinical improvement. Enkephalins are known to modulate inflammatory responses. In the context of psoriasis, the increased levels of enkephalins could be part of the complex inflammatory environment, although their precise role (pro- or anti-inflammatory, or a compensatory mechanism) can be nuanced and depends on the cellular context and receptor interactions. Intradermal injection of Met-enkephalin in normal skin has been shown to induce an inflammatory reaction involving histamine release, while in delayed-type hypersensitivity, it seemed to downregulate the reaction, suggesting context-dependent effects. Proenkephalin expression in the skin can be upregulated by stressful stimuli, including UVB irradiation. This suggests that the increased enkephalin levels in psoriatic skin might also be a response to the inflammatory stress of the disease. UVA irradiation, in particular, has been shown to significantly stimulate enkephalin levels in the skin [61,62,63,64].

### 3.5. Local Corticosteroidogenesis and Its Role in the Pathogenesis of Psoriasis

CYP11A1, also known as P450scc or the cholesterol side-chain cleavage enzyme, is the rate-limiting enzyme in the biosynthesis of steroid hormones. In the skin, CYP11A1 initiates local steroidogenesis by producing steroids directly within skin cells, a process that is distinct from systemic steroid production in the adrenal glands.

In addition to its role in steroid hormone synthesis, CYP11A1 also metabolizes vitamin D3, converting it into several bioactive derivatives not typically produced through systemic metabolism. These include lumisterol (a biologically active isomer of vitamin D) and intermediates derived from 7-dehydrocholesterol (7-DHC), the precursor to vitamin D3 in the skin.

Through these pathways, CYP11A1 contributes to skin homeostasis, supporting barrier integrity, immune regulation, and protection against environmental stressors, including UV radiation and microbial invasion, while also helping to prevent excessive transepidermal water loss [65].

CYP11A1 modulates local immune responses, playing a key role in the skin’s defense against pathogens and in the regulation of inflammation. In psoriasis, its expression is significantly reduced, indicating a potential impairment of local steroidogenesis and altered vitamin D metabolism in psoriatic skin [66,67]. The decreased CYP11A1 activity, leading to reduced local steroid production (including glucocorticoids), contributes to a deficient feedback mechanism involving POMC and glucocorticoids on cutaneous immunity. In a healthy state, glucocorticoids (locally produced or systemically derived) help to suppress inflammatory responses. POMC-derived peptides (like ACTH and α-MSH) also have immunomodulatory roles, often acting to counteract inflammation. If CYP11A1 activity is low, resulting in lower local glucocorticoid levels, the skin’s ability to self-regulate and dampen excessive immune responses is impaired. This deficiency allows inflammatory processes to become chronic or exacerbated. This deficient feedback loop is proposed to contribute directly to the pathogenesis of inflammatory and autoimmune skin conditions like psoriasis. The skin’s immune system becomes overactive without proper endogenous regulation. The restoration of these endogenous deficiencies (i.e., boosting local steroidogenesis and the effects of POMC/glucocorticoids) is seen as a “realistic target” for treating psoriasis and other inflammatory skin disorders. This could involve approaches that directly enhance CYP11A1 activity in the skin; supply precursors that can be converted by remaining CYP11A1; administer specific steroids or vitamin D metabolites that are deficient; or target pathways that compensate for the lack of these endogenous regulatory molecules. In essence, CYP11A1 is a central player in maintaining skin homeostasis, particularly regarding barrier function and immune regulation. Its reduced expression in psoriasis disrupts crucial local regulatory mechanisms, contributing to inflammation, and suggests that restoring its function or compensating for its deficiency could be a viable therapeutic strategy [68,69,70].

Defective glucocorticoid signaling contributes significantly to the pathogenesis of psoriasis and arises from a broad dysregulation of the skin’s local steroidogenic machinery, including reduced expression of key enzymes (CYP11A1, CYP17, 11βHSD1, 11βHSD2), transport proteins (StAR, MLN64), and even the glucocorticoid receptor itself. The consequence is an impaired ability of the skin to locally produce and utilize its own anti-inflammatory glucocorticoids, leading to uncontrolled inflammation and contributing to the characteristic pathology of these diseases. Glucocorticoids are known to be potent anti-inflammatory agents. They act by blocking the production of IL-4 and IL-5, which are pro-inflammatory cytokines, particularly important in Type 2 immune responses. Blocking their production would reduce inflammation [71].

11βHSD1 and 11βHSD2 are enzymes which regulate the interconversion of active and inactive glucocorticoids within tissues. Their reduction implies an impaired ability to control local glucocorticoid levels. A decrease in the glucocorticoid receptor (GR) itself means that even if some glucocorticoids are present, the cells are less responsive to their anti-inflammatory signals. StAR (Steroidogenic Acute Regulatory protein) and MLN64 are proteins that are essential for the transport of cholesterol into the mitochondria—the rate-limiting step in steroidogenesis—and both of these proteins are catalyzed by CYP11A1. Their reduction further compromises the initial steps of steroid production [67].

Tiala et al. reported that CCHCR1, a gene involved in steroidogenesis and vitamin D metabolism, is downregulated in psoriasis. This adds another layer of evidence for impaired steroid and vitamin D pathways [72].

### 3.6. Ultraviolet B and Its Role in Psoriasis

The multifaceted response of the skin to UVB radiation leads to an abnormal impact on the POMC system and its associated peptides and receptors like *α*-MSH [73,74], *β*-endorphin, and ACTH. This response is presumed to serve protective and regulatory functions [73,75,76,77]. Additionally, UVB also boosts CRH and urocortin synthesis [78,79,80] and alters the pattern of CRH receptor type 1 (CRH-R1) expression and activity [81,82,83]. The effect could lead to the disruptions in cellular or molecular interactions within the skin and, finally, could be a cause of psoriasis [66,70,84,85,86,87,88,89].

### 3.7. Vitamin D Signaling Role in Psoriasis

The activation pathways and biological functions of two novel vitamin D3-related compounds—lumisterol 3 (L3) and tachysterol 3 (T3)—are discussed here. Traditionally, vitamin D3 activation is associated with specific hydroxylases. Cytochrome P450 enzymes (CYP11A1 and/or CYP27A1) are identified as initiators of the activation of L3 and T3. The result of these enzymes on L3 and T3 produces various hydroxylated forms, which are the biologically active compounds. Hydroxymetabolites of L3 and T3 demonstrate a wide range of beneficial effects on skin health and disease, including: protection against DNA damage and oxidative stress; stimulation of keratinocyte differentiation; anti-inflammatory activities; antifibrogenic and anticancer activities; and inhibition of cell proliferation [90].

According to mechanisms of action, these hydroxymetabolites exert their effects by interacting with a variety of signaling pathways and receptors like nuclear receptors (such as the Vitamin D Receptor (VDR), the aryl Hydrocarbon Receptor (AhR), LXRα/β (Liver X Receptors), the RAR-related orphan receptor α/γ (RORα/γ), and the peroxisome proliferator-activated receptor-γ (PPAR-γ)). These hydroxymetabolites also exert their effects on signaling pathways such as: the activation of NRF2; activation of p53; inhibition of NF-κB; inhibition of IL-17; and the inhibition of Shh (Sonic hedgehog inhibition of Wnt/β-catenin signaling) [90].

To sum up, by acting through these diverse mechanisms, the hydroxymetabolites of L3 and T3 contribute to maintaining the healthy structure and function of the skin, offering potential for new therapeutic strategies in dermatology [90].

## 4. Conclusions

Visfatin (NAMPT), elafin, and chemerin are promising candidates for both diagnostic applications and as therapeutic targets in immune-mediated, genetic, environmentally influenced, and inflammatory skin diseases such as psoriasis, primarily due to their dual pro- and anti-inflammatory properties. In addition to these emerging proteins, recent evidence highlights the importance of the skin neuro–immuno–endocrine axis, including the role of stress-related neuropeptides, UVB-induced pathways, and vitamin D metabolism, all of which interact with immune and inflammatory mechanisms in psoriasis.

Although preclinical and early clinical studies suggest that these proteins are generally safe and well tolerated, larger clinical trials are essential to assess their long-term efficacy, safety, and therapeutic value. Moreover, further research is needed to clarify optimal diagnostic strategies, delivery routes, dosing, and timing for therapeutic application.
